# Identification of mutations in *BMP15* and *GDF9* genes associated with prolificacy of Markhoz goats

**DOI:** 10.5194/aab-62-565-2019

**Published:** 2019-10-14

**Authors:** Hourad Ghoreishi, Sadegh Fathi-Yosefabad, Jalal Shayegh, Abolfazl Barzegari

**Affiliations:** 1 Department of Animal Sciences, Tabriz Branch, Islamic Azad University, Tabriz, Iran; 2 Department of Veterinary Medicine, Shabestar Branch, Islamic Azad University, Shabestar, Iran; 3 School of Advanced Medical Sciences, Research Center for Pharmaceutical Nanotechnology, Tabriz University of Medical Sciences, Tabriz, Iran

## Abstract

The Markhoz is a local goat breed in the Kurdistan region of Iran. The mohair obtained from these animals plays an important cultural role and is used for making local clothes in the Kurdistan region. This breed is a low-fecundity local goat, and searching for genes associated with fertility of these goats is important for their breeding. Moreover, this research is complementary to prior studies of candidate genes associated with fertility. The growth differentiation factor 9 (*GDF9*) and bone morphogenetic protein 15 (*BMP15*) are attractive candidates expressed by the oocyte and are associated with increased ovulation rate in sheep. However, there are no reports on single nucleotide polymorphisms associated with fertility of Markhoz goats. Hence, we studied these candidate genes and found two novel mutations (g.233C>A and g.755T>G) in *GDF9* exon I and in *BMP15* exon II, respectively. Furthermore, we investigated their association with prolificacy. These nucleotide changes are detectable with the PCR-RFLP method and can be used in the screening for highly fecund goats. Both of the mutations significantly increased litter size in heterozygote form for *BMP15* and homozygote form for *GDF9* in this goat breed. Homozygote females for the *BMP15* mutation were not identified in the Markhoz breed, which is similar to the situation found in Belclare sheep, small-tailed Han sheep, and Jining Grey goats.

## 1 Introduction

Markhoz (Iranian Angora) goats are raised in the Kurdistan region of Iran, and the mohair obtained from these goats has an essential cultural value and is used for making local clothes in Kurdistan, Iran. These small ruminants are effortless and withstand harsh conditions (Farshad et al., 2008). The Markhoz goats live in various arid and semi-arid areas, where they are handled as beneficial animals for the production of milk, meat, hair, and hide. It is a low-fecundity local goat breed. Therefore, it is advantageous to find any genes that can be used in breeding and thus increasing the fecundity.

On the other hand, *GDF9* and *BMP15* genes have been proved to be effective in increasing the number of twin births in the sheep (Abdoli et al., 2013; Barzegari et al., 2010; Liandris et al., 2012; Eghbalsaied et al., 2012); hence, the mutations associated with fecundity in goats should be confirmed (Ahlawat et al., 2013; Pramod et al., 2013). The influence of these genes on fertility in mammals and even in humans may appear progressively. Various candidate genes such as *KiSS-1*, *TSHB*, *POU1F1*, *GPR54*, and *BMPR-IB* have been reported for litter size in goats (Cao et al., 2010, 2011; Chu et al., 2007a; Feng et al., 2012; Huang et al., 2015). However, evidence shows the high influence of *BMP15* and *GDF9* genes on the fecundity (Chu et al., 2010; Feng et al., 2012).

*BMP15* and *GDF9*, two members of the transforming growth factor-β (TGF-β) superfamily, are the key genes involved in increasing the ovulation rate (Knight and Glister, 2006). These genes are produced by the ovary and show intense effects (Knight and Glister, 2006). Moreover, these two oocyte-specific factors induce mitosis along with differentiation in the follicular somatic cells during follicular development through a paracrine signaling pathway (Paulini and Melo, 2011). They are also found to play pivotal roles in specifying ovulation rate and litter size (Galloway et al., 2000; Hanrahan et al., 2004).

Several mutations in these growth factors likely contribute to the high prolificacy such as high ovulation rate or litter size and hence are necessary for ewe reproductivity (Hanrahan et al., 2004). These changes are required for follicular growth, and in addition both of these oocyte-derived growth factors influence ovulation rate in sheep (Hanrahan et al., 2004). Juengel et al. (2004) hypothesized that, in the regulation of ovulation rate in sheep, either *BMP15* and *GDF9* homodimers have essential but similar roles, that *BMP15*/*GDF9* heterodimers have crucial roles, or both. This suggests that both *GDF9* and *BMP15* may play critical roles in not only regulating ovulation rate but in oocyte health together with the establishment of pregnancy (Hanrahan et al., 2004). The equal outcomes are observed in ewes, heterozygous for the Inverdale gene, in which ovulation rate increases lead to a predictable increase in litter size (Yan et al., 2001). The physiological relevance of the synergistic effects of *BMP15* and *GDF9* mutations is emphasized by the observation that ewes, the compound heterozygotes for both *BMP15* and *GDF9* mutations, have significantly higher ovulation rates than heterozygous carriers of a mutation in only one of them.

In this study, SNP of *BMP15* and *GDF9* genes, which plays a very important role in the regulation of folliculogenesis as well as the control of ovulation rate, was detected in a low-fecundity Markhoz (Iranian Angora) goat breed. The association between these genes and prolificacy in these goats were also analyzed.

## 2 Materials and methods

### 2.1 Experimental animals and DNA extraction

Approximately 10 mL of blood was drawn aseptically from the jugular vein of 70 Markhoz does, using EDTA as an anticoagulant. Genomic DNA was extracted from whole blood by the phenol-chloroform method, then dissolved in TE buffer (10 mmol = L Tris HCl and 1 mmol = L EDTA, pH 8.0), and kept at -20 ${}^{\circ }$C.

For the 70 Markhoz does, kidded in 2010–2012, there are data on litter size at the first, second, third, or fourth parity, and the does were chosen randomly from Sanandaj Markhoz Goat Breeding Station, Kurdistan region of Iran. No selection on litter size or other fertility traits was made in the flock over previous years. The average age and litter size of the does were 38.7 months and 1.25, respectively. These animals were under the full supervision of the Kurdistan Agricultural Jihad Organization and under the standard conditions of management, health, and nutrition.

### 2.2 Amplification of *BMP15* and *GDF9* genes and sequencing

The primers were designed using Oligo-7 software for exon I of the *GDF9* and exon II of the *BMP15* genes from goat genomic *BMP15* and *GDF9* sequences that were recorded in NCBI GenBank (*BMP15*, EU743938.1; *GDF9*, EU883989.1). PCR amplification was performed using a 25 µL reaction mixture, containing 1 U Taq DNA polymerase (CinnaGen Co. nos. 2 and 7, Iran), 0.2 µM of each primer, and 40 ng of caprine genomic DNA. The cycling program was set as follows: initial denaturation at 94 ${}^{\circ }$C for 4 min, followed by 35 cycles of denaturation at 94 ${}^{\circ }$C for 50 s; annealing at 61 ${}^{\circ }$C for *GDF9* and 59 ${}^{\circ }$C for *BMP15* for 40 s; extension at 72 ${}^{\circ }$C for 25 s, and a final extension at 72 ${}^{\circ }$C for 10 min. The sequences of primers, restriction enzymes, and PCR reaction conditions are described in Table 1. In order to distinguish any possible mutations in exons of these two genes, the PCR products from the samples of prolific and non-prolific females were randomly sequenced. Then, PCR-RFLP was used to confirm the mutations that appeared in gene sequencing.

**Table 1:** Primers and PCR conditions of the genes and restriction enzymes.

Gene	No.	Primer sequence (5${}^{\prime }$ → 3${}^{\prime }$)	Annealing	Restriction enzyme
			temperature	
*GDF9* (exon I)	F1	GAAGACTGGTATGGGGAAATG	61 ${}^{\circ }$C	BsaI
463 bp	R1	CCAATCTGCTCCTACACACCT		
*BMP15* (exon II)	F2	CAGTTTGTACTGAGCAGGTC	59 ${}^{\circ }$C	PsiI
857 bp	R2	TTTGCCGTCACCTGCATGTG		

### 2.3 PCR-RFLP analysis

The exon I of the *GDF9* and exon II of the *BMP15* genes, from some sequenced samples, had mutations in nucleotide no. 233 with C>A type and in nucleotide no. 755 with T>G type and contained XmiI (AccI) and BpuJI restriction sites, respectively. The PCR products were digested with 0.5 µL of Eco31I (BsaI) enzyme for *GDF9* exon I and AanI (PsiI) enzyme for *BMP15* exon II (Fermentase Co.) overnight at 37 ${}^{\circ }$C and the resulting products were separated by 2 % agarose gel electrophoresis. Although both mutations were silent, statistical analysis was performed to find out whether or not they affect prolificacy in this breed.

### 2.4 Statistical analyses

Analysis of variance of litter size data undergoing the least square procedures was conducted for *GDF9*, *BMP15*, and their combined genotypes. Therefore, the following model was fitted to compare differences in litter size among different genotypes:

1 $$y=\mu +\mathrm{KS}+P+G_{1}+G_{2}+G_{1}G_{2}+s+e,$$

where y is the litter size phenotypic value; μ is the population mean; KS is the fixed kidding season effect; P is the fixed parity effect; $G_{1}$ is the fixed effect for *GDF9* genotypes; $G_{2}$ is the fixed effect for *BMP15* genotypes; $G_{1}G_{2}$ is the fixed interaction effect for *GDF9* and *BMP15* combined genotypes; s is the random sire effect; and e is the random error effect of each observation. The variation among does within genotypes was used to calculate standard errors. The analysis was performed using the general linear model (GLM) procedure of SAS (Ver. 8.1) (SAS Institute Inc., Cary, NC, USA). Mean separation procedures were performed using the least significant difference test.

## 3 Results

### 3.1 Sequence results and novel SNP detection

**Figure 1:**
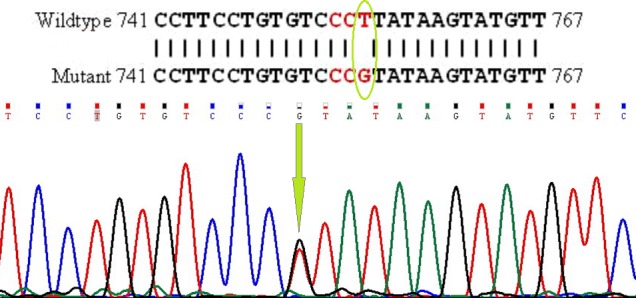
Partial sequence of *BMP15* (exon II) and T to G transition in heterozygote goat.

**Figure 2:**
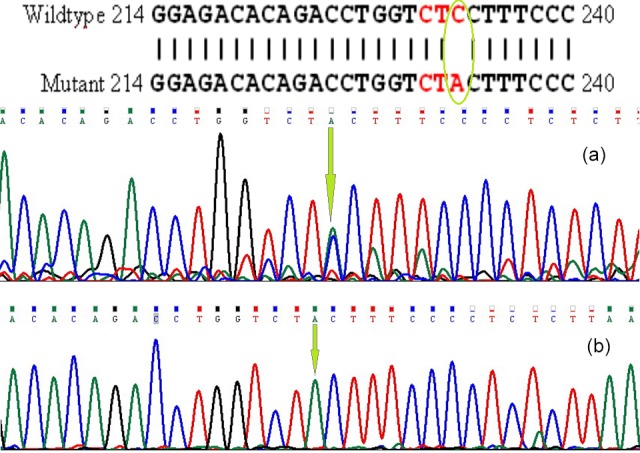
Nucleotide change in *GDF9* (exon I): **(a)** heterozygote goat, **(b)** homozygote goat.

The PCR products were electrophoresed in 1 % agarose gel for *BMP15* and *GDF9* genes. The products were then sequenced by Macrogen Inc. (South Korea). Afterward, using BLAST software (http://blast.ncbi.nlm.nih.gov/Blast.cgi, last access: 15 January 2010), the determined sequences were aligned with the *Capra hircus* nucleotide database in NCBI GenBank. They were submitted with accession numbers of GU732196.1 and GU784823.2 for *BMP15* and *GDF9*, respectively. The loci of the mutations are shown in Figs. 1 and 2.

### 3.2 Determination of PCR-RFLP and statistical analyses

**Figure 3:**
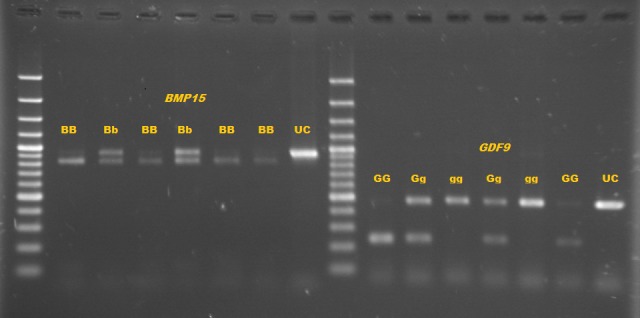
Genotypes of *BMP15* gene, BB (757 bp/100 bp), and Bb (857 bp/757 bp/100 bp) (UC - uncut). Genotypes of *GDF9* gene, GG (234 bp/229 bp), Gg (463 bp/234 bp/229 bp), and gg (463 bp).

PCR-RFLP products are displayed in Fig. 3. Three genotypes of GG (234 bp/229 bp), Gg (234 bp/229 bp/463 bp), and gg (463 bp/463 bp) were detected for *GDF9* gene, while only two genotypes, BB (757 bp/100 bp) and Bb (857 bp/757 bp/100 bp), were identified for *BMP15* gene (Fig. 3). These mutations result in no change in the amino acid codes that are Leucine and Proline for *GDF9* and *BMP15*, respectively (Table 2). Allelic and genotypic frequencies of the mutations of *GDF9* and *BMP15* genes are presented in Table 3.

**Table 2:** The novel single nucleotide polymorphism in *BMP15* and *GDF9* within the Markhoz.

Gene	Base	Coding	Coding	Amino acid change
	change	base	residue	
		(bp)	(aa)	
*BMP15*	T-G	1083	361	Unchanged Pro (P)
*GDF9*	C-A	183	61	Unchanged Leu (L)

**Table 3:** Allele and genotype frequencies of the *GDF9* gene and *BMP15* gene.

No. of does	Allele frequency		Genotype frequency		
70	G	g	GG	Gg	gg
	0.54	0.46	0.280	0.520	0.200
	B	b	BB	Bb	bb
	0.847	0.153	0.693	0.307	0.0

For the *GDF9* gene, frequencies of genotypes GG, Gg, and gg were 0.28, 0.52, and 0.2, respectively. While for the *BMP15* gene, frequencies of genotypes BB, Bb, and bb were 0.69, 0.31, and 0.00, respectively. Combined genotypic frequencies of *GDF9* and *BMP15* mutations are presented in Table 4. The highest and the lowest frequencies for combined genotypes were 0.37 and 0.05 for GgBB and GGBb, respectively.

**Table 4:** Combined genotypic frequencies of *GDF9* and *BMP15* genes.

No. of does	Genotype frequency					
70	GGBB	GGBb	GgBB	GgBb	ggBB	ggBb
	0.227	0.053	0.373	0.147	0.093	0.107

Litter size in Markhoz goats was significantly influenced by *GDF9* (P=0.029) and *BMP15* (P=0.0307) genotypes. No significant interaction was detected among *GDF9* and *BMP15* genotypes. Moreover, the least square means and standard error for litter size of different *GDF9* and *BMP15* and their combined genotypes in Markhoz goats are given in Table 5. Does with the heterozygote mutant Bb genotype had 0.231 (P<0.05) more kids than those with the wild type BB genotype. The Markhoz does with homozygote mutant gg genotype had 0.597 (P<0.01) and 0.491 (P<0.01) more kids than those with wild type GG and heterozygote mutant Gg genotypes, respectively. Does with mutations in both *GDF9* and *BMP15* genes had greater litter size than those with each mutation alone. Similarly, the effect of the *GDF9* gene mutation was greater than that of the *BMP15* gene mutation on litter size in the combined genotypes of Markhoz does.

**Table 5:** Least square means and SE for litter size of different *GDF9* and *BMP15* genotypes.

Genotype${}^{*}$	No. of observations	Litter size
*GDF9* genotypes		
GG	19	$1.052^{B}\pm 0.158$
Gg	36	$1.158^{B}\pm 0.132$
gg	15	$1.649^{A}\pm 0.164$
*BMP15* genotypes		
BB	48	$1.171^{b}\pm 0.121$
Bb	22	$1.402^{a}\pm 0.136$
Combined genotypes		
GGBB	15	$0.871^{\mathrm{Bd}}\pm 0.157$
GGBb	4	$1.233^{b}\pm 0.211$
GgBB	26	$1.130^{\mathrm{Bd}}\pm 0.127$
GgBb	10	$1.186^{B}\pm 0.172$
ggBB	7	$1.511^{c}\pm 0.202$
ggBb	8	$1.788^{\mathrm{Aa}}\pm 0.180$

${}^{\text{A–B}}$ Least square means with different letters within each of the three sets (*GDF9*, *BMP15*, or combined genotypes) different at P<0.01. ${}^{\text{a–d}}$ Least square means with different letters within each of the three sets (*GDF9*, *BMP15*, or combined genotypes) different at P<0.05. ${}^{*}$ g - *GDF9* mutation; b - *BMP15* mutation; G and B - wild type.

## 4 Discussion

Some mutations have been demonstrated to be involved in twin births along with fecundity increase in two *GDF9* and *BMP15* genes in sheep (Barzegari et al., 2010; Galloway et al., 2000; Hanrahan et al., 2004; Javanmard et al., 2011). The reported mutations in sheep remain dissociated with the fecundity in goats (Godara et al., 2012); however, the mutations in other positions in two genes of goats imply the prominent influence of these two genes on fecundity of mammals.

This article constitutes introductory research concerning the possible prolific mechanisms in goats. The present study reports two new nucleotide polymorphisms in the goat *GDF9* and *BMP15* genes, with no amino acid change in the mature coding region of the peptide. PCR-RFLP with BsaI and PsiI digestion was used to investigate the polymorphism of exon I of *GDF9* and exon II of *BMP15* genes, respectively. The PCR products were separated by 1 % agarose gel electrophoresis, and the following digestion with restriction enzymes was separated by 2 % agarose gel electrophoresis (Fig. 3). Both of these mutations were associated with increased litter size in Markhoz goat. In other words, litter size was significantly altered by *GDF9* and *BMP15* genotypes.

Does carrying one copy of *BMP15* mutation had more kids than those with the wild type genotype. No homozygote mutant genotype was detected for *BMP15* gene. A potential reason is that the mutation is an inactivating mutation in homozygote form; hence females carrying two copies of this inactivating mutation are sterile and are likely removed from the flock. There have been no substantial reports on infertility among Markhoz does until now. Seemingly, no bb genotype females live in the Markhoz breed, similar to the situation that was obtained for Belclare sheep, small-tailed Han sheep, and Jining Grey goats (as a hypothesis) (Chu et al., 2007b; Hanrahan et al., 2004). Controlled breeding of Bb genotype bucks and does and large-scale research, studying the effect of bucks on litter size and investigating more breeds, are required to confirm this hypothesis.

The Markhoz does with two copies of the *GDF9* mutation had more kids than those with the wild type and those carrying mutations in one copy of the mutation. There was no significant difference between does with heterozygote mutation of *GDF9* and does with wild type genotype. Does with mutations in both *GDF9* and *BMP15* genes had greater litter size than those with one copy of each of the mutations. Similarly, the effect of the *GDF9* gene mutation was greater than that of the mutation of the *BMP15* gene on litter size in the combined genotypes of Markhoz does.

### Ethical issues

No animal or human studies were carried out by the authors.

## Data Availability

The data that support the findings of this study are available from the Kurdistan Agricultural Jihad Organization, but restrictions apply to the availability of these data, which were used under license for the current study, so they are not publicly available. Data are however available from the authors upon reasonable request and with permission from the Kurdistan Agricultural Jihad Organization.
